# Glucocorticoids Modulate Expression of Perineuronal Net Component Genes and Parvalbumin During Development of Mouse Cortical Neurons

**DOI:** 10.1007/s12035-025-05369-4

**Published:** 2025-11-29

**Authors:** Liang Yue, Michael T. Craig, Brian J. Morris

**Affiliations:** School of Psychology and Neuroscience, College of Medical, Veterinary and Life Sciences, University of Glasgow, Sir Joseph Black Building, Glasgow, G12 8QQ UK

**Keywords:** Neurocan, Mifepristone, RU486, Has3, Bral2, Non-genomic

## Abstract

Severe prenatal maternal stress is a risk factor for schizophrenia in offspring. Since parvalbumin-containing GABAergic interneuron function in the cortex and hippocampus is compromised in schizophrenia, and perineuronal nets (PNNs) facilitate the functioning of these cells, we tested the hypothesis that glucocorticoids, as stress mediators that can access the foetal compartment, might influence the expression of PNN component genes. In cultured mouse cortical neurons, we detected effects of hydrocortisone on many PNN component genes, via diverse mechanisms. A rapid (< 4 h), glucocorticoid receptor (GR)-mediated suppression of *neurocan* and hyaluronan synthase (*Has*) 1 and 3 mRNAs was observed at 7 days in vitro (DIV), whereas at 14 DIV, *brevican* and *versican* expression was reduced by hydrocortisone without GR involvement, while GR inhibition elevated *Has1* and *Has2* mRNA levels and suppressed *aggrecan* mRNA levels. *Tenascin R* expression was rapidly suppressed by hydrocortisone at 7 DIV but not at 14. At 21 DIV, PNN component gene expression had become insensitive to hydrocortisone, although *parvalbumin* expression was reduced after 24 h but not 4 h exposure. Additionally, effects on protein levels were observed that were sometimes consistent with the mRNA changes (e.g. Has3, Gad1) and sometimes unrelated to them (e.g. elevated TnR levels at 7 DIV after glucocorticoid receptor antagonism). We found that hydrocortisone could directly inhibit proteasome activity. As expected from these results, the overall structure of the PNN was compromised by hydrocortisone exposure, with the length of the proximal dendrite covered by PNN being reduced. Overall, the data demonstrate a complex and profound, but developmental stage-dependent, regulation of PNN component gene expression by glucocorticoids. This may contribute to the action of severe prenatal or perinatal stress to increase schizophrenia risk.

**Supplementary Information:**

The online version contains supplementary material available at 10.1007/s12035-025-05369-4.

## Introduction

Perineuronal nets (PNNs) are complex structures composed of extracellular matrix molecules, covering the somata, dendrites and proximal axon segments of distinct neuronal populations [1], mainly enveloping fast-spiking GABAergic parvalbumin (*Pvalb*)-expressing interneurons in cortical and hippocampal areas [2]. Their formation in the developing brain is associated with the onset of *Pvalb* expression, the maturation of the *Pvalb*-expressing cells, and the closure of the critical period of cortical plasticity [3–5]. Once formed, the PNNs are believed to enable fast-spiking activity of these cells by restricting ion movements immediately extracellular to the cell membrane [1, 6], while also protecting the cells from oxidative stress [3] and consolidating afferent synapse architecture [7].

The key components of PNNs are the members of the family of chondroitin sulphate proteoglycans (CSPGs), including aggrecan (*Acan*), brevican (*Bcan*), neurocan (*Ncan*), versican (*Vcan*) and phosphacan (*Ptprz1*), which are essential for PNN function and structure [Deepa et al., 2006]. Hyaluronan (HA), which forms the backbone of PNNs, binding to the CSPGs, is synthesised by cell membrane hyaluronan synthase (Has) enzymes, and extruded into the extracellular space. The structure is stabilised by hyaluronan and proteoglycan link proteins (Haplns), and by cross-linking by Tenascin R [1]. The cellular origins of these different components are not always clear, but it is likely that some are synthesised in neurons and others in astrocytes [8, 9], suggesting that a complex regulatory framework must be required for effective coordination of the synthesis of the different constituent proteins in different cells.

During development, PNNs are susceptible to external stressors. Reduced PNN staining intensity and density were found in various brain areas, including prefrontal cortex (PFC), basolateral amygdala [10, 11] and hippocampus [12] in rodents after exposure to early life stress. Consistent evidence has also demonstrated downregulated levels of CSPG molecules after exposure to stress [13, 14].

PNNs are disrupted as part of the dysfunction of *Pvalb*-expressing neurons in schizophrenia. Alongside the reduced expression of *PVALB* and the GABA synthetic enzyme *GAD1*/GAD67 in PFC, hippocampus and thalamic reticular nucleus in schizophrenia [15, 16], PNN structure is also compromised [17–20]. Maternal experience of severe stress (such as famine or natural disaster) during pregnancy increases the risk of schizophrenia for the offspring in utero [21]. While the causal mechanisms are unclear, it is known that glucocorticoids (GCs), which are markedly elevated during the maternal stress response, can cross the placenta and access the foetal compartment [22–25]. Considering the reports that early life stress could influence PNN development, we considered the hypothesis that GCs might affect the expression of PNN component genes, and hence disrupt the formation or maintenance of PNNs and increase schizophrenia risk.

In humans, the main GC is cortisol, while in rodents, the main GC is corticosterone. The canonical GC mechanism involves binding to an intracellular receptor, either the GC receptor (GR) or mineralocorticoid receptor (MR), nuclear translocation of the steroid-receptor complex, and binding to specific sites, usually in gene promoter regions, resulting in repression or activation of gene expression [26]. These transcriptional effects are slow – a typical time course would have an onset of altered mRNA levels 8–12 h after GC exposure [26, 27], although occasionally more rapid effects can be observed. The altered levels would then be maintained for more than 24 h after the original exposure. The non-genomic effects of GCs, which are thought to involve membrane receptor-mediated actions, not necessarily involving alterations in gene transcription, occur much more rapidly (< 1 h) [26, 28–30]. There is also evidence that some rapid, non-genomic effects of GCs do not involve the canonical GR, but rather other receptors such as GPR97 (*ADGRG3*) located on the cell membrane [31].

Thus, we sought to test the hypothesis that elevated GC levels would disrupt PNN formation in developing neuronal networks, and to uncover the underlying mechanisms.

## Methods

### Primary Cortical Neuronal Culture

Mouse neurons were cultured as previously described [32, 33]. Cortical cultures were prepared from pooled cortical tissue from 3–6 E17 mouse embryos from the same litter. The brains were removed from C57BL/6 mouse embryos at E17 and washed in ice-cold Hanks Balanced Salt Solution (HBSS). The meninges were removed, the cortical tissues were isolated and transferred into clean ice-cold HBSS. After two more washes in clean ice-cold HBSS, the cortical tissues were transferred into 0.05% trypsin/EDTA (Gibco, 25300054) at 37 °C for 10 min. Following this, DMEM (10% HI horse serum, 1% Penicillin-Streptomycin, 1% Glutamax) was added to inactivate the trypsin and the tissues were centrifuged at 1500 rpm for 5 min. After removing the supernatant, DMEM (8 ml/embryo) was added, and the mixture of DMEM and cortical tissues was transferred into clean neurobasal medium (Gibco, 21103049) with B27 supplement (Gibco, 17504044). The cells were then seeded into 12-well plates precoated with 4 μg/ml poly-D-lysine and 6 μg/ml laminin. Subsequently, neurobasal medium with B27 supplement and clean DMEM medium were added to each well. The ratio of cell suspension medium, clean neurobasal medium and DMEM medium in each well was 0.25/0.25/0.5. After 24 h, 50% medium in each well was replaced with new neurobasal medium with B27 supplement; and then 50% of the medium in each well was changed with Neurobasal/B27 every 4 days for the duration of the cultures.

Note that these primary neuronal cultures contain only a small proportion of astrocytes (~ 3–5% in our hands, as assessed by glial fibrillary acidic protein vs tubulin immunocytochemistry), due to the maintenance conditions designed to suppress astrocyte survival.

### Drug Treatment

To investigate the effect of glucocorticoids on PNN expression at 7, 14 and 21 days in vitro (DIV), cells were treated with low dose (final concentration 20 nM) or high dose (final concentration 100 nM) hydrocortisone acetate (CORT) for 4 h or 24 h. These concentrations were chosen to reproduce those achieved in the foetus during moderate or severe maternal emotional stress (see “Discussion”). In some experiments, the selective GR antagonist, mifepristone/RU486 (20 nM final concentration) was added 30 min prior to CORT treatment. In addition, to examine the pharmacology of GC effects, the selective MR agonist, aldosterone (100 nM final concentration), or the selective GR agonist, fluticasone (50 nM final concentration), were tested. Alternatively, cells were also treated with collagen 3 (Cell Guidance Systems, 75 nM final concentration), an agonist ligand for the adhesion GPCRs GPR56/97 [34–36], to investigate whether the effect of glucocorticoids was mediated through this GPCR family. To test whether mRNA stability was affected, CORT treatment occurred together with 5 µg/ml Actinomycin D treatment, to inhibit the mRNA synthesis, 1.5 h, 2 h, 3 h, or 4 h prior to mRNA extraction.

### mRNA Extraction, cDNA Synthesis and Quantitative Polymerase Chain Reaction (qPCR)

The procedure was according to our standard methodology [37, 38]. Total mRNA was isolated from the cultured cells using RNeasy mini kits (Qiagen 74106). The RNA quality and concentration were confirmed using spectrophotometry before cDNA synthesis. First strand cDNA was synthesised from mRNA using high-capacity RNA-to-cDNA kit with 10 µl RT Buffer (Applied biosystem, 4387406) and 1 µl enzyme (Applied biosystem, 4387406), in a final volume of 20 μL with appropriate volumes of nuclease-free water and mRNA samples based on the results of RNA spectrophotometry. The product was aliquoted and stored at −20 °C for future use. The cDNA quality and concentration were confirmed using spectrophotometry before the qPCR method.

mRNA levels were measured using SYBRgreen methodology. Samples were run in triplicate on 96-well plates with 1 μl of cDNA samples, 19 μl master mix (Agilent), with cycling conditions of 1 cycle at 50 °C for 2 min, 1 cycle at 95 °C for 2 min, 40 cycles for 30 s at 95 °C and 10 s at 60 °C, followed by a melt curve. *Gapdh* was employed as the housekeeping gene to normalise the gene expression of different targeted primers, except in a single case where the treatment (collagen 3) was found to alter *Gapdh* expression, where *Tbp* was used instead. The primers targeted *Acan*, *Bcan*, *Ncan*, *Vcan*, *Ptprz1*, *Has1*, *Has2*, *Has3*, *TnR*, *Hapln4*, *Gad1*, *Gad2* and *Pvalb*. Data were analysed using the ΔΔCt method. Primer sequences are provided in Supplementary Table 1.

### Protein Extraction and Western Blot

Immunoblotting was performed via our standard procedures [39, 40]. The medium was removed from the wells and 1 ml ice-cold PBS (pH 7.4) was added to each well for about 1 min. Following this, 60 μl RIPA buffer (made up with 50 mM Tris–HCl, 150 mM NaCl, 1% Triton 100, 0.15% SDS, 0.5% sodium deoxycholate and 50 ml dH2O) with 1% protease inhibitor cocktail (Sigma P-8340) and 1% sodium orthovanadate was added to the wells for 3 min. The wells were then scraped with pipette tips, the contents were transferred to 1.5 ml Eppendorf tubes and centrifuged for 10 min at 4 °C, 13,000 rpm. Supernatants were collected and the protein concentration was measured using Bradford Protein Assay [Bradford, 1976]. The protein samples (−1.5 μg/μl) were prepared with 4× sample buffer (NuPAGE, Novex, NP0007) and reducing reagent (NuPAGE, Novex, NP0004). Protein samples were denatured at 80 °C for 10 min, and 25 μl/lane was added to SDS-PAGE in 4%–12% Bis–Tris gels (NuPAGE, Novex, NP0302BOX), followed by electrophoresis at 200 V for 1.5 h in chilled running buffer.

Protein was then transferred to Invitrogen PVALBDF membranes (Invitrogen, LC2002) in transfer buffer at room temperature running for 1 h at 30 V. Membranes were washed twice in ddH2O and blocked in 0.5% Tween-Tris-buffered saline (TTBS) with 3% dried milk powder (Marvel) for 30 min at room temperature. After blocking, membranes were incubated with primary antibodies at 4 °C overnight in 1% TTBS milk. The following morning, membranes were washed 3 times for 10 min in Tris-buffered saline (TBS) containing 0.05% Tween 20 (Sigma-Aldrich, T7949) and incubated in horseradish peroxidase (HRP)-conjugated anti-mouse/anti-rabbit secondary antibodies (anti-mouse concentration: 1:10,000; anti-rabbit concentration: 1:6000) with 1% dried milk in TTBS for 1.5–2 h at room temperature. The antibodies that were used in western blots are listed in Supplementary Table 2.

Membranes were then washed once with TTBS and washed twice with TBS after incubation of secondary antibodies. Membranes with targeted antibody could be detected by adding chemiluminescent HRP Substrate (Immobilon, Millipore, WBKLS0100) using equal quantities of luminol and peroxide solution. Finally, the membranes were placed into a cassette and images were captured using PXI4 (Syngene) with varied exposure times depending on the antibodies used.

### Lectin Fluorescence

Wisteria floribunda agglutinin (WFA) lectin is widely used as a marker to visualise PNNs [41]. For immunofluorescence, cells were fixed at 14 DIV with 4% paraformaldehyde for 30 min. Cells were then permeabilised and blocked with PBS (0.3 M NaCl)/0.25%Triton X-100/10% normal goat serum (NGS) for 1 h at room temperature. Following the blocking step, cells were incubated in a humidified chamber with biotinylated WFA diluted in 0.3 M PBS/0.25%Triton X-100/3% NGS at 4 °C overnight. On the following day, cells were incubated in streptavidin-conjugated Rhodamine Red-X diluted in 0.3 M PBS/3% NGS for 1 h in the dark. After incubation in the dark, cells were washed three times in 0.3 M PBS and mounted with Vectashield mounting media (Vector Laboratories, H-1200). The slides were finally covered with a coverslip.

Images were scanned using a confocal microscope (ZEISS, LSM900) with a 10× objective for counting and a 20× objective for presentation. All representative images were captured as a Z-stack (15 µm in depth) using a Z step of 0.50 µm, 20× objective lens, image size 1024 × 1024 pixels. The images were taken using Zen blue 3.0 software and downloaded with summed intensity zen-stack projection in the Zen black system. Image analyses were performed using ImageJ [42]. The PNN-covering dendrite length and intensity were measured manually in ImageJ.

### Proteasome Activity

All 3 catalytic 20S proteasome activities were measured by detecting the 7-amino-4-methylcoumarin (AMC) fluorescence liberated from the synthetic proteasomal substrates: Suc-Leu-Leu-Val-Tyr-AMC (Suc-LLVY-AMC) for chymotrypsin-like activity, Boc-Leu-Ala-Ala-AMC (Boc-LAA-AMC) for trypsin-like activity and Ac-Glu-Pro-Leu-Asp-AMC (Ac-GPLD-AMC) for caspase-like activity. To recognise whether the activities were related to the proteasome, MG132 was used as an inhibitor for all 3 activities of the 20S proteasome. Unconjugated AMC was diluted into appropriate concentrations to create a standard curve which allowed the fluorescence signal to be converted to units of AMC. The released fluorescence was measured every 60 s for 30 min using an excitation wavelength of 340 nm and an emission wavelength of 450 nm.

### Statistical Analysis

Statistical analysis was conducted using Minitab, with ANOVA as the standard approach after checking for normality of data distribution, with Tukey or Fisher post hoc tests depending on the *F* value of the factor in ANOVA. Sample sizes given represent the number of individual culture wells from which mRNA or protein was extracted separately. Samples were nested within the culture from which they derived for statistical analysis.

## Results

Efficiency of amplification was tested for all primer pairs, and was in every case between 90 and 120% (Supplementary Information Figure S1). In addition, preliminary experiments determined that the expression of *Gapdh* mRNA was not affected by the treatments, consistent with previous studies in the laboratory [38], and was therefore an appropriate choice for “housekeeping gene” in these studies, apart from collagen 3 exposure, where *Gapdh* mRNA levels were affected by the treatment but *Tbp* mRNA levels did not change, and so it was used instead (data not shown).

### CORT Treatment Altered the mRNA Expression of PNN Components During Neuronal Development Period

The present study aims to investigate the effect of glucocorticoids on the gene expression of PNN components in mouse cortical neurones. The components tested in the study included CSPGs (*Acan*, *Bcan*, *Ncan* and *Vcan*), HAS (*Has1*, *Has2* and *Has3*), *Hapln4* (also known as Brain link protein 2/Bral2) and tenascin R (*TnR*). Attempts were also made to detect *Hapln* 1, *Hapln2* and *Hapln3*, but they appeared to be below the threshold for reliable detection (Ct’s ~ 33 or higher).

We then studied the effect of exposure at 7, 14 and 21 DIV to low (20 nM) or high dose (100 nM) CORT and where effects of CORT were observed, we sought to investigate the involvement of GR activation in the actions: mifepristone (20 nM) was co-treated with CORT. Mifepristone has nanomolar affinity for glucocorticoid receptors, but micromolar affinity for mineralocorticoid receptors [Testas et al., 1983; Galanuad et al., 1984; Cain et al., 2019].

A summary table of the key findings (Table 1) is provided at the end of the Results section.

**Table 1:** Summary effect of glucocorticoid and mifepristone effects. ↓: tendency to decrease after CORT or collagen3; ↑ tendency to increase after CORT or collagen3; ↓* decrease was statistically significant; ↑* increase was statistically significant; ^#^overall significant effect of mifepristone, ^#^*p* < 0.05, ^##^*p* < 0.01; ^###^*p* < 0.001

	7 DIV						14 DIV						21 DIV					
	HCA				Mif		HCA				Mif		HCA				Mif	
	4 h low	4 h high	24 h low	24 h high	4 h	24 h	4 h low	4 h high	24 h low	24 h high	4 h	24 h	4 h low	4 h high	24 h low	24 h high	4 h	24 h
*Acan*	↓	↓			^#^		↓	↓										
*Bcan*							↓*	↓*		↓	^#^							
*Ncan*	↓*		↓	↓		^###^												
*Vcan*								↓*										
*Pcan*																		
*Has1*	↓	↓				^#^						^###^						
*Has2*	↓	↓										^##^						
*Has3*		↓*																
*Hapln4*			↓	↓			↓*											
*TnR*	↓*	↓*		↑*		^#^												
*Pvalb*					^#^											↓*		
*Gad1*	↓			↓				↓*										
*Gad2*																		

**Fig. 1:**
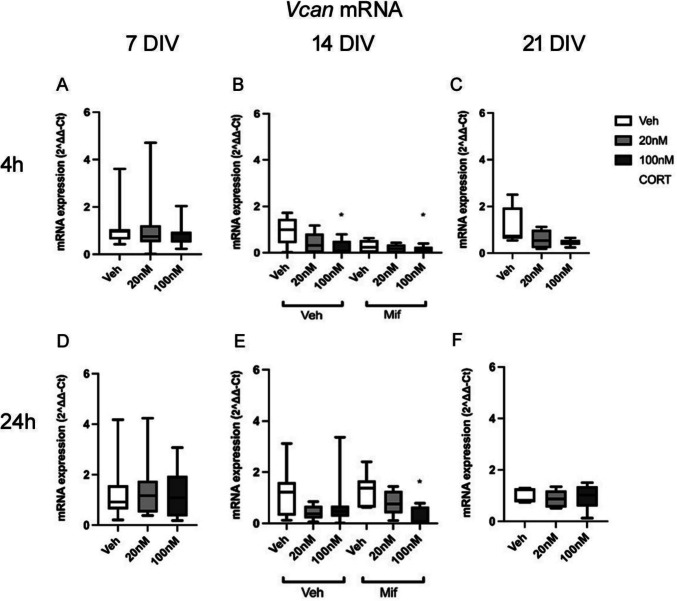
**A**–**F** mRNA expression of *Vcan* after 4 h and 24 h exposure to low dose (20 nM) and high dose (100 nM) CORT at 7, 14 and 21 DIV, at 7 and 21 DIV with CORT alone, and at 14 DIV also with mifepristone (20 nM) treatment. Sample numbers: **A**, **D**—*n* = 14–16/group; **B**–**F**—*n* = 7–8/group. *p < 0.05 vs corresponding vehicle group, post hoc Tukey’s test. Boxes show median and interquartile range, with whiskers from minimum to maximum

The mRNA expression of *Vcan* was not significantly changed by CORT exposure at 7 DIV (4 h: *F*(2,43) = 1.02, *p* = 0.370; 24 h: *F*(2,42) = 0.42, *p* = 0.66) (Fig. 1 A, D), or 21 DIV (4 h: *F*(2,19) = 3.00, *p* = 0.085; 24 h: *F*(2,18) = 0.11, *p* = 0.894) after 4 h and 24 h CORT treatment (Fig. 1 C, F).

However, clear changes were observed at 14 DIV. There was a significant effect of CORT treatment at both 4 h (*F*(2,46) = 3.32, *p* = 0.046) and 24 h (*F*(2,46) = 3.92, *p* = 0.028) (Fig. 1 B, E). The post hoc testing revealed that the expression of *Vcan* decreased after high dose CORT treatment at both 4 h and 24 h relative to vehicle treatment (4 h: veh vs high dose CORT *p* = 0.048, 24 h: veh vs high dose CORT *p* = 0.021). The overall effect of mifepristone, independent of whether CORT was absent or present, was not significant at 4 h (*F*(2,46) = 2.37, *p* = 0.131) or 24 h (*F*(2,46) = 0.01, *p* = 0.943). However, the interaction of CORT and mifepristone was significant at 4 h (*F*(2,46) = 4.63, *p* = 0.016) (Fig. 1B), but not at 24 h (*F*(2,46) = 0.43, *p* = 0.653) (Fig. 1E), suggesting that mifepristone treatment exacerbated the corticosterone-driven suppression of *Vcan* expression at 4 h (e.g. veh/mifepristone vs veh/veh—*p* = 0.027 Fisher post hoc test). Hence, glucocorticoids seem to exert a rapid non-GR-mediated suppression of *Vcan* mRNA levels, and a basal glucocorticoid receptor-mediated enhancement by GCs in the medium, which is rapidly blocked by mifepristone. In short, a rapid suppressive non-GR action and a rapid enhancing GR action on *Vcan* expression.

No significant effect of CORT on *Vcan* expression was observed at 21 DIV (Supplementary figure S3).

**Fig. 2:**
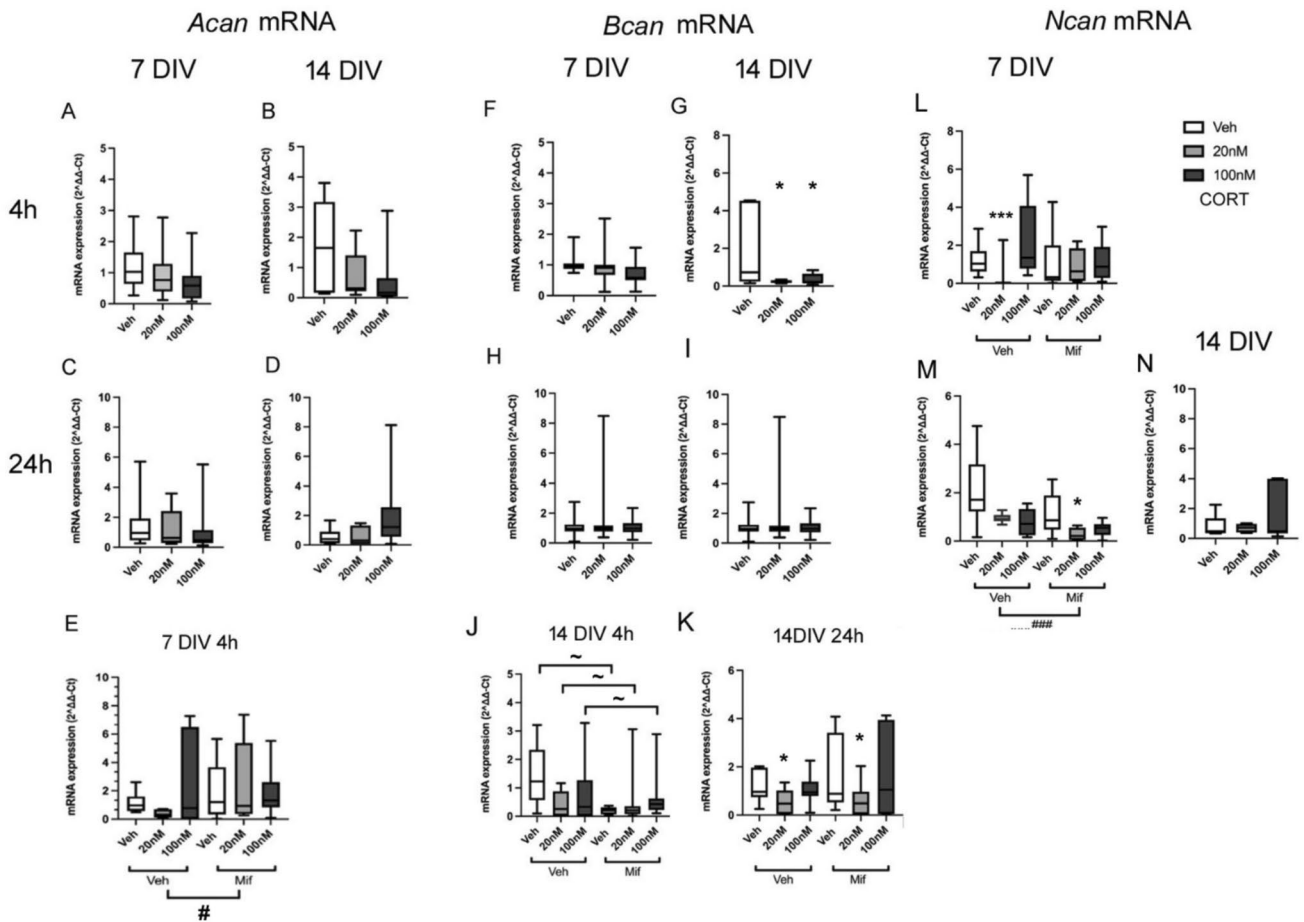
Effect of glucocorticoid exposure on Acan, Bcan and Ncan mRNA expression. mRNA expression of Acan (**A**–**E**), Bcan (**F**–**K**), or Ncan (**L**–**N**) after exposure to vehicle (Veh), low dose (20 nM) or high dose (100 nM) CORT at 7 (**A**,** C**,** E**,** F**,** H**,** L**,** M**) or 14 (**B**,** D**,** G**,** I**,** J**,** K**,** N**) DIV, with drug exposure for 4 h (**A**,** B**,** E**,** F**,** G**,** J**,** L**) or 24 h (**C**,** D**,** H**,** I**,** K**,** M**,** N**). 7 DIV experiments with CORT only, *n* = 14–16/group; experiments also with mifepristone (20 nM), *n* = 7–8/group; 14 DIV experiments with CORT only, *n* = 6–8/group; experiments also with mifepristone (20 nM), *n* = 7–8/group; 21 DIV *n* = 6–8/group. ^#^*p* < 0.05, ^###^*p* < 0.001 overall effect mifepristone vs vehicle groups (ANOVA); *p < 0.05, ***p < 0.001 vs corresponding vehicle group, ^~^*p* < 0.05 for comparison shown (post hoc Tukey test). Boxes show median and interquartile range, with whiskers from minimum to maximum

There was a tendency for *Acan* mRNA expression at 7 DIV to decrease after high dose CORT treatment at 4 h (*F*(2,43) = 3.06, *p* = 0.058; high dose VS vehicle, *p* = 0.046, post hoc Tukey test), but not 24 h (*F*(2,47) = 1.32, *p* = 0.277) (Fig. 2 A, C). No significant effects were detected at 14 (Fig. 2 B, D) or 21 DIV (Supplementary figure S3) (14 DIV 4 h: *F*(2,19) = 2.11, *p* = 0.152, 24 h: *F*(2,21) = 2.11, *p* = 0.152; 21 DIV 4 h: *F*(2,18) = 1. 06, *p* = 0.370, 24 h: *F*(2,19) = 3.49, *p* = 0.085), although the same trend for suppression at 4 but not 24 h was noted at 14 DIV.

When the experiment was repeated to test any influence of mifepristone, no significant effect of CORT was shown with 4 h exposure (*F*(2,44) = 0.25, *p* = 0.779) (Fig. 2 E), although there was a hint of a suppression as before (*p* = 0.085 veh without mifepristone vs 20 nM CORT without mifepristone, post hoc Tukey test), but mifepristone overall significantly increased expression of *Acan* (*F*(2,44) = 4.18, *p* = 0.048). The interaction of mifepristone and CORT treatment was not significant (*F*(2,44) = 1.78, *p* = 0.183). The results suggested that there was some basal suppression of *Acan* expression by GCs, which limited the ability to detect further suppression with CORT application, but which was revealed by mifepristone exposure.

*Bcan* mRNA expression decreased significantly after 4 h exposure to low dose and high dose CORT treatment at 14 DIV (*F*(2,19) = 3.62, *p* = 0.048; veh vs low dose: *p* = 0.025, veh vs high dose *p* = 0.035, Fisher post hoc tests), despite considerable variability in the control group (Fig. 2 G). No significant changes in gene expression were detected at 7 DIV (4 h: *F*(2,43) = 2.80, *p* = 0.072; 24 h: *F*(2,47) = 0.85, *p* = 0.436) (Fig. 2 F), 14 DIV (24 h: *F*(2,21) = 0.53, *p* = 0.596)(Fig. 2I) or 21 DIV (4 h: *F*(2,18) = 1.06, *p* = 0.704) (Supplementary Fig. S3). While the effects of CORT at 14 DIV were only marginally significant, the effects appeared genuine, as when the experiment was repeated in the absence or presence of mifepristone, *Bcan* mRNA levels were again found to be decreased by CORT in the absence of mifepristone (veh vs low dose CORT *p* = 0.039, veh vs high dose CORT *p* = 0.020, Fisher post hoc tests). There was an overall significant effect of CORT treatment on *Bcan* mRNA expression at 14 DIV after 24 h (*F*(2,46) = 6.85, *p* = 0.003), but not 4 h (*F*(2,46) = 1.19, *p* = 0.316), suggesting that the mRNA expression of *Bcan* decreased significantly after 24 h low dose CORT treatment (veh vs low dose CORT *p* = 0.002 Tukey post hoc tests) (Fig. 2J, K). The results did not show a significant overall effect of mifepristone on *Bcan* expression, at either 4 h (*F*(2,46) = 0.29, *p* = 0.592) or 24 h (*F*(2,46) = 0.97, p = 0.329). These results indicated that at 14 DIV, the ability of 20 nM CORT to reduce *Bcan* mRNA levels was very clearly unaffected by the presence of mifepristone (Fig. 2J, K). There was a significant interaction of CORT and mifepristone treatment at 4 h (*F*(2,46) = 3.63, *p* = 0.036), but not 24 h (*F*(2,46) = 0.51, *p* = 0.603). Mifepristone caused a suppression of *Bcan* mRNA levels (veh with mifepristone vs veh without mifepristone *p* = 0.018, Fisher post hoc tests). At 21 DIV, the expression of *Bcan* was not affected by CORT at 4 h (*F*(2,44) = 0.59, *p* = 0.560) or 24 h (*F*(2,44) = 2.78, *p* = 0.075). Moreover, the effect of mifepristone was not significant either, at 4 h (*F*(1,44) = 0.01, *p* = 0.936) or 24 h (*F*(1,44) = 1.58, *p* = 0.217). Similarly, the interaction of mifepristone and CORT treatment was not significant with 4 h (*F*(1,44) = 1.24, *p* = 3.000) and 24 h (*F*(1,44) = 0.78, *p* = 0.466) exposure (Supplementary Fig. 3).

Hence, just as with the regulation of *Vcan* expression, GCs seem to exert a rapid non-GR-mediated suppression of *Bcan* mRNA levels by CORT. These complex effects on *Bcan* mRNA suggest a rapid non-GR-mediated suppression by CORT, and a basal GR-mediated enhancement by GCs in the medium, which is rapidly blocked by mifepristone at 14 DIV.

CORT exposure also showed an ability to suppress *Ncan* expression. At 7 DIV, a significant effect of CORT on *Ncan* was detected after 4 h (Fig. 2L) (*F*(2,45) = 7.66, *p* = 0.002) and 24 h (*F*(2,45) = 4.96, *p* = 0.012) (veh vs low dose CORT at 4 h *p* = 0.008) and decreased with both low and high dose treatment at 24 h (veh vs low dose CORT *p* = 0.017, veh vs high dose CORT *p* = 0.036, Tukey post hoc tests). The same reduction was also detected in the presence of mifepristone, with lower expression after 24 h low dose CORT (veh vs low dose CORT, *p* = 0.023, Tukey post hoc tests), but no significant changes with 4 h CORT exposure in the presence of mifepristone (veh vs low dose CORT, *p* = 0.869, veh vs high dose CORT, *p* = 0.683, Tukey post hoc tests). The interaction of mifepristone and CORT treatment was significant after 4 h (*F*(2,45) = 9.79, *p* < 0.001), and 24 h (*F*(2,45) = 2.20, *p* = 0.124), in that the expression of *Ncan* with 4 h low dose CORT exposure was increased in the presence of mifepristone compared to in its absence (*p* = 0.024, Tukey post hoc tests), but decreased for the same comparison after 24 h (*p* = 0.006). Additionally, there was an overall effect of mifepristone to decrease *Ncan* mRNA expression over 24 h (*F*(2,45) = 12.47, *p* = 0.001), but not 4 h (*F*(2,45) = 1,06, *p* = 0.310). Overall this suggests a suppressive effect of slightly increasing GC levels at 4 h through GRs (mifepristone sensitive), and also not via GRs over a longer time scale (mifepristone insensitive), combined with the basal levels of GCs in the medium tending to enhance *Ncan* expression (probably genomic in mechanism, since slowly relieved over 24 h by mifepristone, resulting in a further decrease in levels). No changes in Ncan expression were detected at 21 DIV (Fig. 2 O, Supplementary Fig. 3).

**Fig. 3:**
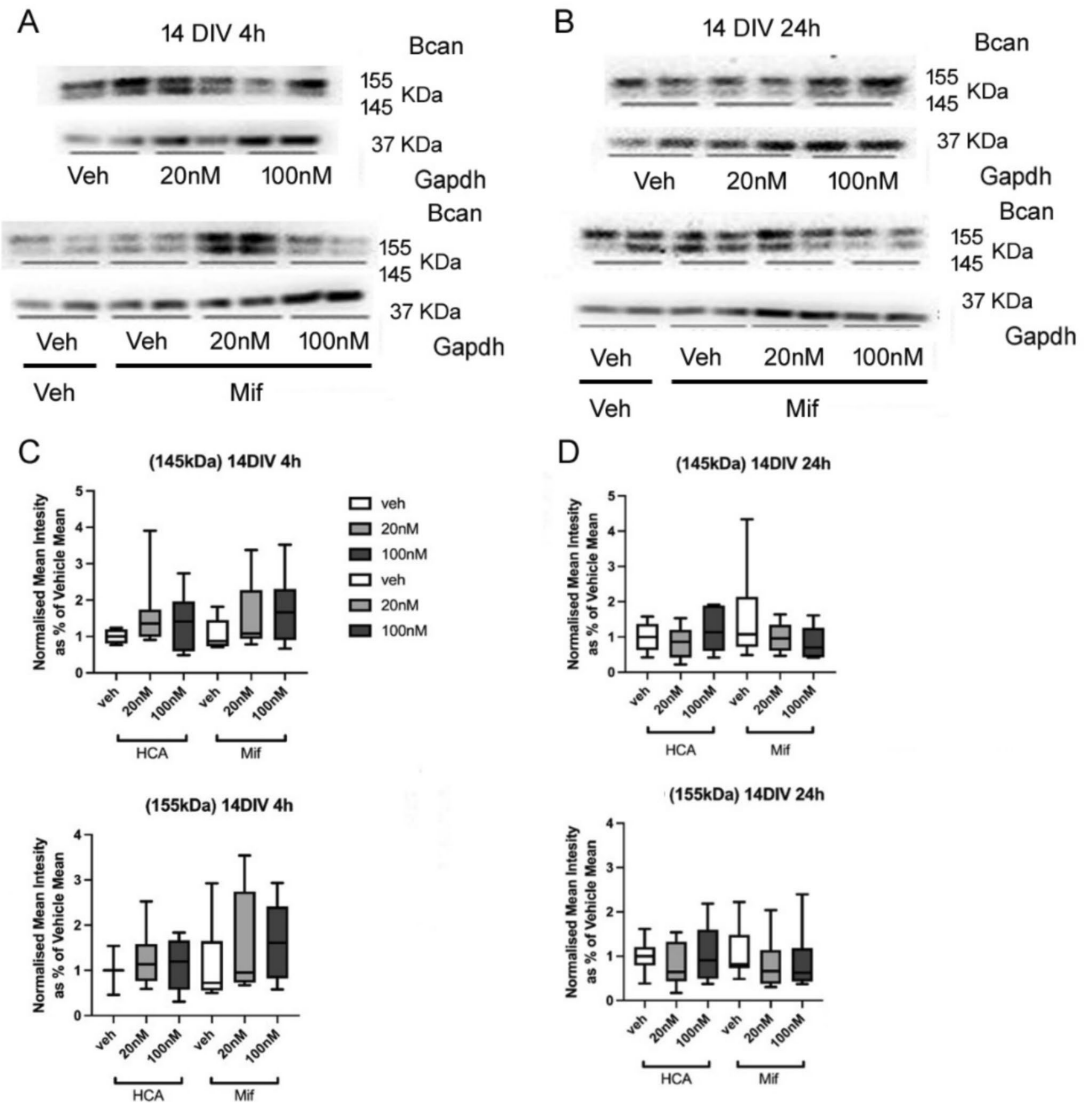
Bcan protein expression after exposure to CORT (20 or 100 nM) and/or mifepristone (20 nM). Representative results from western blotting are shown after 4 h (**A**) or 24 h (**B**) exposure at 14 DIV (**C**, **D**) corresponding box and whisker plots showing normalised mean intensity of the band signals after CORT and mifepristone exposure at 14 DIV with 4 h (**C**) or 24 h (**D**) exposure, for immunoreactive bands at 145 and 155 KDa (*n* = 46 in total, Veh: veh = 7, 20 nM = 8, 100 nM = 8; Mifepristone: veh = 7, 20 nM = 8, 100 nM = 8). Boxes show median and interquartile range, with whiskers from minimum to maximum

Although several *Bcan* mRNA alterations were observed at 14 DIV, the protein expression of *Bcan* (155 kDa) (Fig. 3 A C) (4 h: *F*(2,26) = 1.5, *p* = 0.227) remained unchanged after 4 h CORT exposure. With mifepristone co-treatment, no significant changes were observed (Fig. 3 A—D) (155 kDa/145 kDa) protein levels with both 4 h and 24 h CORT exposure (155 kDa: 4 h: *F*(2,47) = 0.97, *p* = 0.389, 24 h: *F*(2,47) = 0.77, *p* = 0.469; 145 kDa:4 h: *F*(2,47) = 2.64, *p* = 0.083, 24 h: *F*(2,47) = 1.10 *p* = 0.342). Moreover, there were no overall effects of mifepristone (155 kDa: 4 h: *F*(1,47) = 1.54, *p* = 0.221, 24 h: *F*(1,47) = 0.00, *p* = 0.963; 145 kDa:4 h: *F*(1,47) = 0.21, *p* = 0.652, 24 h: *F*(1,47) = 0.23, *p* = 0.631) and no significant interactions of CORT and mifepristone (155 kDa: 4 h: *F*(2,47) = 0.27, *p* = 0.765, 24 h: *F*(2,47) = 0.28, *p* = 0.759; 145 kDa: 4 h: *F*(2,47) = 0.38, *p* = 0.686, 24 h: *F*(2,47) = 1.73, *p* = 0.190) detected after 4 h and 24 h exposure.

For *phosphacan* (*Ptprz1*), mRNA expression was unchanged after 4 h or 24 h CORT exposure, with low or high doses, at 7 DIV, 14 DIV and 21 DIV (Supplementary figures S3, S4).

**Fig. 4:**
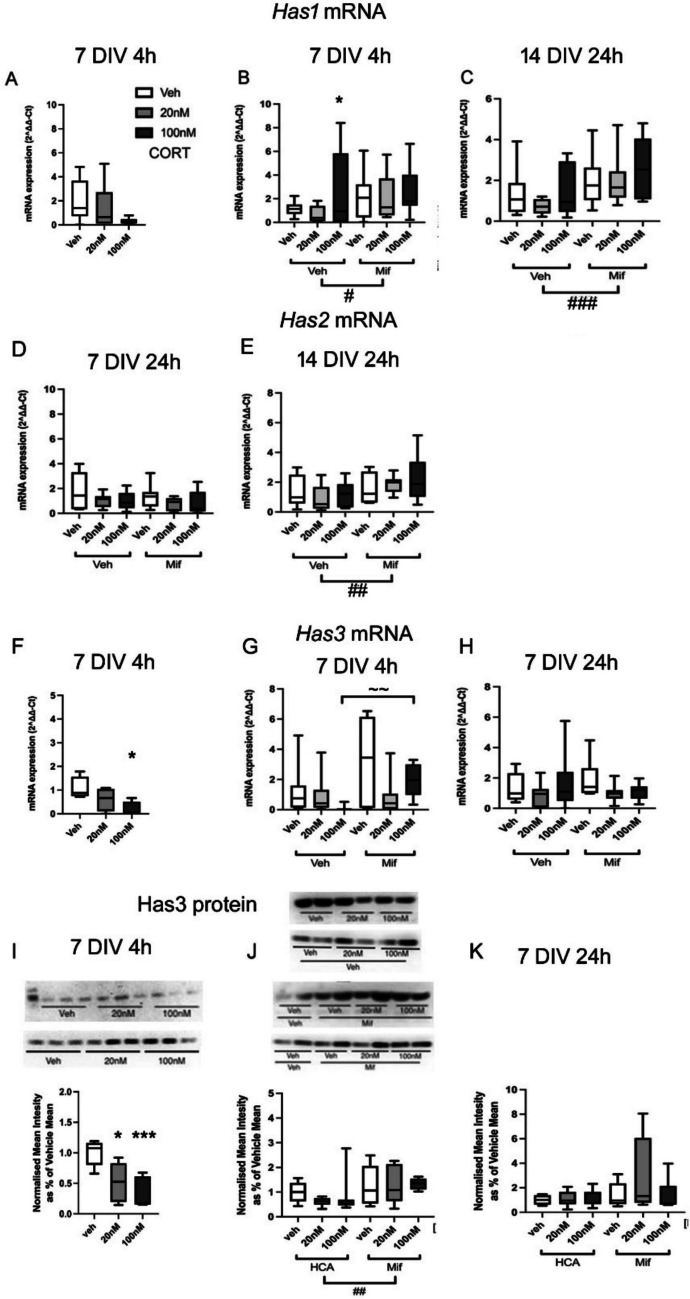
Effects of CORT on Has gene expression **A**–**C**: Has1 mRNA expression; **D**, **E**: Has2 mRNA expression, and **F**–**H**: Has3 mRNA expression after 4 h or 24 h exposure to low dose (20 nM) or high dose (100 nM) CORT at 7 or 14 DIV, in the absence or presence of mifepristone (20 nM) (7 DIV, *n* = 6–8/group; 14 DIV, *n* = 7–8/group); **I**, **J**: Western blot analysis of Has3 protein levels at 7 DIV after 4 h exposure to 20 nM or 100 nM CORT alone (**I**), or without or with mifepristone (20 nM) treatment (**J**). *n*: veh = 8, 20 nM = 8, 100 nM = 8; Mifepristone: veh = 8, 20 nM = 8, 100 nM = 8. Example blots are shown, with corresponding quantification below. **K**: Corresponding western blot analysis of Has3 protein levels exposed to the same doses of CORT for 24 h. *n*: veh = 12, 20 nM CORT = 12, 100 nM CORT = 10. *p < 0.05, ***p < 0.001 vs corresponding vehicle group, post hoc Tukey’s test; ^~~^*p* < 0.01 for comparison shown, post hoc Fisher’s test; ^#^*p* < 0.05, ^##^*p* < 0.01, ^###^*p* < 0.001, overall effect of mifepristone vs corresponding (mifepristone) vehicle group, ANOVA. Boxes show median and interquartile range, with whiskers from minimum to maximum

*Has1* gene expression was not significantly affected at 7 DIV (4 h: *F*(2,21) = 2.02, *p* = 0.161; 24 h: *F*(2,23) = 2.71, *p* = 0.09) (Fig. 4 A) or 14 DIV (4 h: *F*(2,8) = 0.07, *p* = 0.933; 24 h: *F*(2,12) = 1.75, *p* = 0.223) (Fig. 4 C and data not shown), although there was a trend towards a decrease in *Has1* expression at 7 DIV after 4 h with 100 nM CORT (*p* = 0.068, post hoc Tukey test). When repeated without or with mifepristone, there was again a tendency towards decreased *Has1* mRNA expression with 4 h CORT exposure to the lower dose (Fig. 4 A, B), but there was also a great deal of variability with the higher dose. Overall, including mifepristone groups, there was no significant CORT effect (*F*(2,44) = 0.204 *p* = 0.959) (Fig. 4 B). There was however an overall effect of mifepristone (*F*(2,44) = 6.75, *p* = 0.014), indicating mifepristone significantly increased expression of *Has1*, consistent with medium GCs acting to suppress mRNA levels*.* The mifepristone x CORT treatment interaction was not significant (*F*(2,44) = 1.78, *p* = 0.183). After long-term (24 h) exposure to CORT and mifepristone at 7 DIV, the mRNA levels of *Has1* still remained unchanged (*F*(2,44) = 1.34, *p* = 0.273), and with no overall effect of mifepristone (*F*(1,44) = 1.29, *p* = 0.263) and no interactions of CORT and mifepristone (*F*(2,44) = 0.26, *p* = 0.775) found (data not shown). At 14 DIV, no significant effect of CORT was observed after 24 h CORT treatment (*F*(2,46) = 0.88, *p* = 0.422), but there was now a really clear overall effect of mifepristone (*F*(2,46) = 12.48, *p* = 0.001) (Fig. 4C). The interaction of mifepristone and CORT treatment was not significant (*F*(2,46) = 0.35, *p* = 0.708). These results suggested that mifepristone significantly blocked a basal suppression of *Has1* expression by glucocorticoids in the culture medium, probably rendering it problematic to reveal a clear additional effect of CORT addition. The results suggested a rapidly relievable effect of glucocorticoids in the culture medium to suppress *Has1* mRNA levels at 7 and 14 DIV, an effect attenuated by mifepristone exposure, and slightly enhanced by exogenous CORT exposure.

For *Has2* mRNA expression, no overall effect of mifepristone was detected at 7 DIV at 4 h (*F*(2,44) = 2.50, *p* = 0.123), with no significant interaction of mifepristone and CORT treatment (*F*(2,44) = 0.80, *p* = 0.457) (data not shown). Equally, no alterations of *Has2* mRNA levels were observed after longer-term exposure to CORT (*F*(2,44) = 1.98, *p* = 0.151) at 7 DIV, and no overall effect of mifepristone was detected either (*F*(1,44) = 1.85, *p* = 0.181) (Fig. 4 D), with no interactions between CORT and mifepristone (*F*(1,44) = 0.38, *p* = 0.688). At 14 DIV, the changes in *Has2* expression were again not significant 24 h after CORT treatment (*F*(2,43) = 0.36, *p* = 0.701), but the expression of *Has2* was significantly affected by mifepristone (*F*(2,43) = 7.20, *p* = 0.011) (Fig. 4 E), suggesting increased *Has2* expression after blocking GRs. Interactions of mifepristone and CORT treatment were not significant (*F*(2,43) = 1.46, *p* = 0.246). At 21 DIV, the effect of CORT was not significant at 24 h (*F*(2,46) = 0.25, *p* = 0.783) (Supplementary figure S3). Mifepristone did not have an overall effect on *Has2* expression at 24 h either (*F*(1,47) = 0.11, *p* = 0.738), and there was no significant interaction of mifepristone and CORT treatment detected with 24 h (*F*(2,46) = 1.10, *p* = 0.303) exposure.

*Has3* mRNA expression decreased significantly at 7 DIV after 4 h 100 nM CORT treatment (*F*(2,9) = 5.40, *p* = 0.038, veh vs high dose CORT, *p* = 0.039, Tukey post hoc test) (Fig. 4 F). With mifepristone, at 7 DIV, while there was no significant effect of CORT treatment overall at either 4 h (*F*(2,45) = 0.82, *p* = 0.452) or 24 h (*F*(2,45) = 1.97, *p* = 0.753) (Fig. 4 G, H), and the overall effect of mifepristone was not significant either, at 4 h (*F*(2,45) = 2.04, *p* = 0.163) or 24 h (*F*(2,45) = 2.45, *p* = 0.126) (Fig. 4 G, H), there was a significant interaction between CORT and mifepristone treatment after 4 h (*F*(2,45) = 3.65, *p* = 0.039), but not 24 h (*F*(2,45) = 0.05, *p* = 0.955), suggesting a tendency for mifepristone to attenuate the suppression of *Has3* mRNA levels by exposure to the high dose of CORT (high dose CORT with mifepristone vs high dose CORT without mifepristone *p* = 0.009, Fisher post hoc test). At 21 DIV, no significant effects of CORT on the expression of *Has3* mRNA levels were found at 4 h (*F*(2,47) = 1.79, *p* = 0.179) or 24 h (*F*(2,47) = 0.24, *p* = 0.787) (Supplementary figure S3). There was no effect of mifepristone at 4 h (*F*(1,47) = 0.30, *p* = 0.43) or 24 h (*F*(1,47) = 2.70, *p* = 0.108). Additionally, no significant interaction of mifepristone and CORT treatment was detected with 24 h (*F*(2,47) = 0.30, *p* = 0.743) exposure (Supplementary figure S3). The results imply that, at 7 DIV, a suppressive effect of CORT on *Has3* mRNA levels is rapidly relieved by antagonism of GRs.

We checked for corresponding protein alterations after CORT exposure; Has3 protein levels decreased significantly after 4 h exposure with both low dose and high dose CORT (*F*(2,17) = 11.14, *p* = 0.014 veh vs low dose CORT, *p* = 0.001 veh vs high dose CORT, Tukey post hoc test) (Fig. 4I). When re-tested with mifepristone, the same trend was observed for CORT in the absence of mifepristone at 4 h but not 24 h exposure, but no overall CORT effect was found at either 4 h (*F*(2,47) = 0.41, *p* = 0.665) or 24 h (*F*(2,47) = 1.21, *p* = 0.309) of CORT and mifepristone exposure (Fig. 4 J, K). However, mifepristone significantly increased *Has3* protein levels after 4 h (*F*(1,47) = 8.29, *p* = 0.006) (Fig. 4 J), but not quite significantly after 24 h exposure (*F*(1,47) = 3.38, *p* = 0.073) (Fig. 4 K). No significant interactions of CORT and mifepristone were observed with either 4 h or 24 h exposure (4 h: *F*(2,47) = 0.51, *p* = 0.603, 24 h: *F*(2,47) = 1.12, *p* = 0.336). The evidence therefore points to a rapid suppression of neuronal *Has3* expression at both mRNA and protein levels by GCs via GRs.

Expression of *Hapln4* mRNA was unaffected by CORT after either dose or treatment time, except for a clear suppression of mRNA levels at 4 h (but not 24 h) after treatment at 14 DIV that was not attenuated by mifepristone (Supplementary figure S5). However, Hapln4 protein levels were unchanged by CORT exposure at 14 DIV (Supplementary figure S5).

**Fig. 5:**
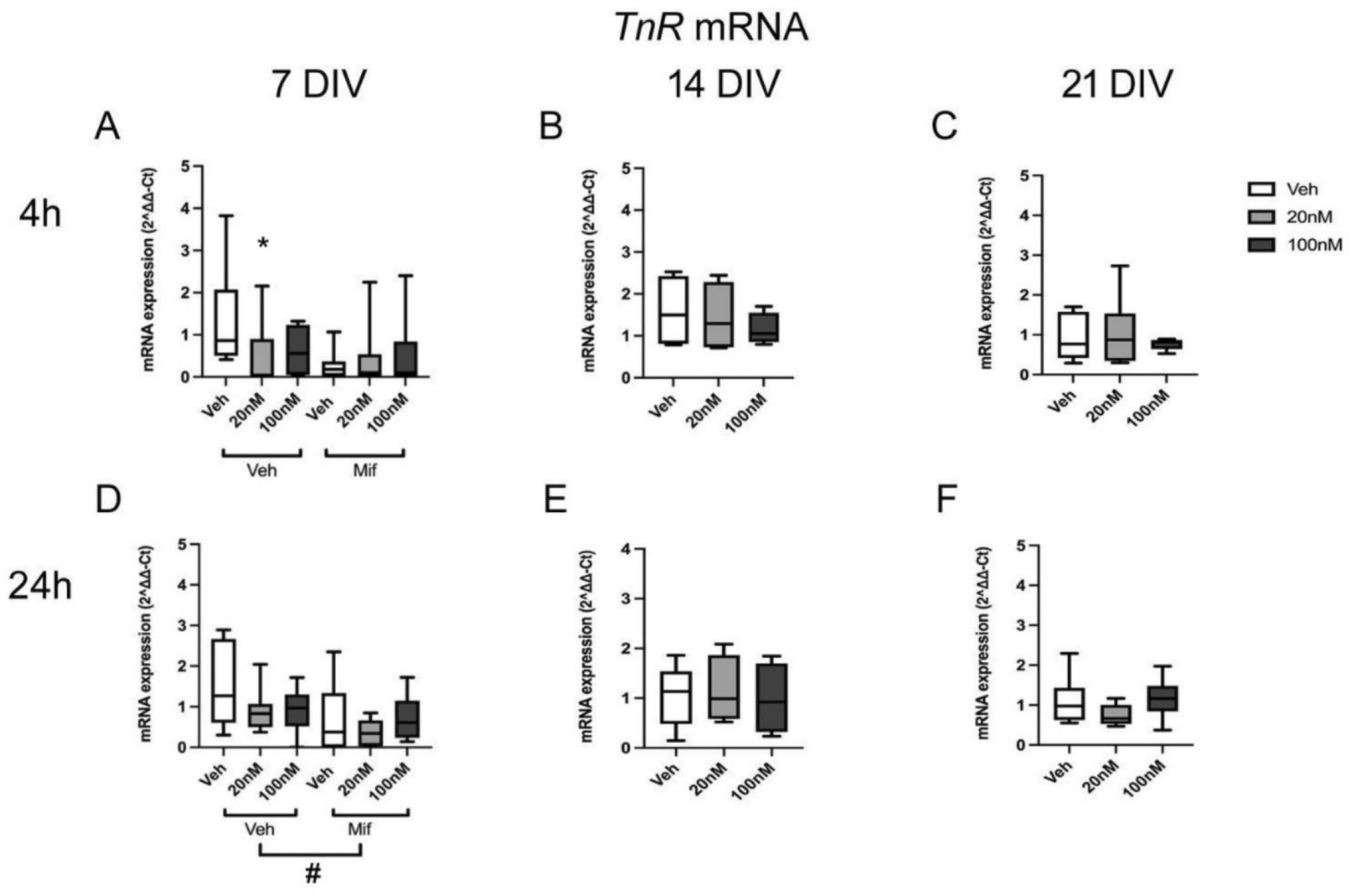
Effect of glucocorticoid exposure on TnR mRNA expression. **A**–**F**: mRNA expression of TnR after 4 h or 24 h exposure to low dose (20 nM) or high dose (100 nM) CORT at 7, 14 and 21 DIV (**B**–**F**), and in the absence or presence of mifepristone (20 nM) (**A**, **D**) (7 DIV: *n* = 7–8/group; 14 DIV: *n* = 9–11/group; 21 DIV: *n* = 12–14/group). ^#^*p* < 0.05, overall effect of mifepristone vs corresponding (mifepristone) vehicle group, ANOVA. Boxes show median and interquartile range, with whiskers from minimum to maximum

The mRNA levels for *TnR* decreased significantly at 7 DIV after 4 h low dose CORT treatment (*F*(2,45) = 3.28, *p* = 0.049, veh vs low dose CORT *p* = 0.038, Tukey post hoc tests), but while the same trend was observed at 24 h, the effect was not significant (*F*(2,45) = 0.49, *p* = 0.619), so the interpretation of the CORT effects remains equivocal. This decrease was maintained in the presence of mifepristone after 4 h CORT exposure, but the mRNA level decreased with the presence of mifepristone with high dose CORT. There was an overall effect of mifepristone on *TnR* expression which was significant after 24 h (*F*(2,45) = 9.17, *p* = 0.004), but not 4 h (*F*(2,45) = 1.69, *p* = 0.201) (Fig. 5 D). The level of mRNA expression with 24 h mifepristone and CORT exposure was lower than that in the absence of mifepristone.

In addition, there was a significant interaction of CORT treatment and mifepristone treatment after 24 h (*F*(2,45) = 5.42, *p* = 0.008) rather than 4 h (*F*(2,45) = 2.51, *p* = 0.095), suggesting a reduction of mRNA levels of the veh treatment group after long-term mifepristone exposure (veh without mifepristone vs veh with mifepristone *p* < 0.001, low dose CORT with mifepristone vs low dose CORT without mifepristone *p* = 0.045, Fisher post hoc tests). There were no significant changes in *TnR* expression after CORT exposure at 14 DIV (4 h: *F*(2,12) = 1.32, *p* = 0.309; 24 h: *F*(2,11) = 0.33, *p* = 0.729) (Fig. 5 B, E) or 21 DIV (4 h: *F*(2,16) = 0.01, *p* = 0.990; 24 h: *F*(2,20) = 1.65, *p* = 0.219) after either 4 h and 24 h treatment (Fig. 5 C, F).

In contrast to the decrease in mRNA expression, protein levels of TnR remained unchanged with 4 h or 24 h CORT exposure at 7 DIV (Supplementary figure S6). However, mifepristone exposure elevated TnR (180 KDa) protein content at 4 h (*F*(1,47) = 5.83, *p* = 0.018) (Supplementary figure S6).

### CORT Alters GABAergic Gene Expression

As PNNs surround mainly Pvalb-expressing GABAergic interneurons [3, 43], GABA-related components, including glutamate decarboxylase (*Gad*) genes and *Pvalb w*ere also measured in the current study.

**Fig. 6:**
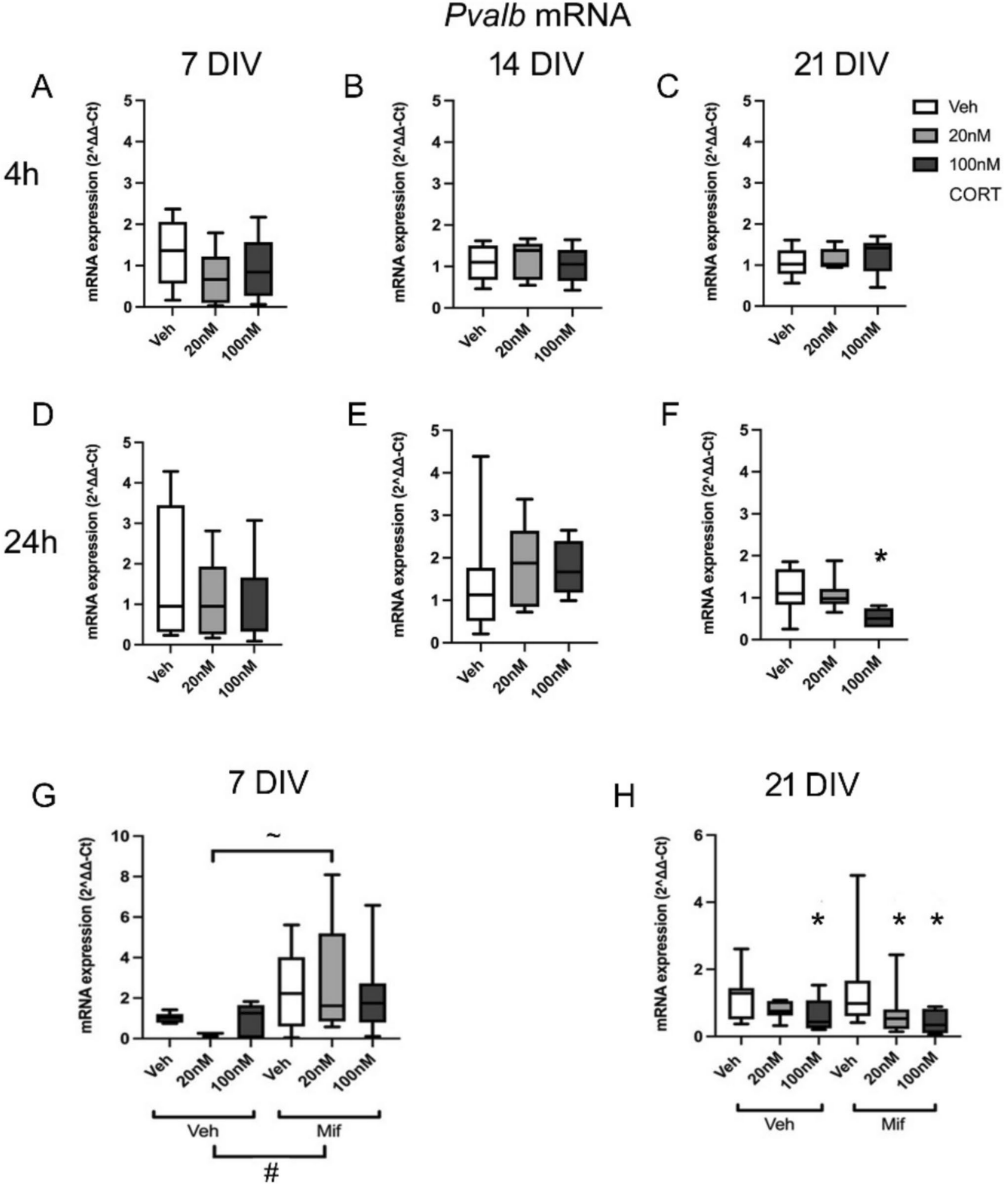
**A**–**F**: mRNA expression of *Pvalb* after 4 h and 24 h exposure to low dose (20 nM) or high dose (100 nM) CORT at 7, 14 and 21 DIV (n=6-8/group). **G**, **H**: mRNA expression of *Pvalb* at 7 DIV (4 h) and 21 DIV (24 h), after CORT and mifepristone (20 nM) treatment (*n* = 45 in total, Veh: veh = 8, low dose samples = 8, high dose samples = 7; Mifepristone: veh = 8, low dose samples = 7, high dose samples = 7) *p < 0.05 vs corresponding vehicle group, ^~^*p* < 0.05 for comparison shown, post hoc Tukey’s test; ^#^*p* < 0.05 overall effect of mifepristone vs corresponding vehicle group, ANOVA. Boxes show median and interquartile range, with whiskers from minimum to maximum

The expression of *Pvalb* decreased significantly after 24 h high dose CORT treatment at 21 DIV (*F*(2,20) = 4.72, *p* = 0.023, veh vs high dose CORT *p* = 0.043, Tukey post hoc tests), but not 4 h (*F*(2,18) = 0.26, *p* = 0.775) (Fig. 6 C, F). However, there were no significant changes at 7 DIV (4 h: *F*(2,23) = 1.25, *p* = 0.308; 24 h: *F*(2,22) = 0.50, *p* = 0.613) (Fig. 6 A, D) or 14 DIV (4 h: *F*(2,23) = 0.25, *p* = 0.785; 24 h: *F*(2,22) = 1.31, *p* = 0.292) (Fig. 6 B, E), after either 4 h or 24 h CORT treatment.

When CORT treatment was repeated, either in the absence or presence of mifepristone, the results again showed that at 7 DIV, there was no significant effect of CORT on the expression of *Pvalb* after 4 h exposure (*F*(2,43) = 0.89, *p* = 0.420) (Fig. 6 G). However, there was a significant overall effect of mifepristone (*F*(1,43) = 12.40, *p* = 0.001) with an increase in the expression of *Pvalb* in cultures exposed to mifepristone. For individual group comparisons, the *Pvalb* mRNA levels significantly increased with 4 h exposure by 20 nM CORT with mifepristone compared to 20 nM CORT without mifepristone (*p* = 0.022, Tukey post hoc tests), with the same trend at other CORT doses. This implies a basal GR-mediated suppression of *Pvalb* mRNA at 7 DIV (despite the low basal levels of expression at this developmental stage). At 21 DIV, there was once more a significant effect of high dose CORT after 24 h exposure, maintained in the presence of mifepristone (*F*(2,47) = 8.66, *p* = 0.001; veh with mifepristone vs low dose CORT with mifepristone *p* = 0.035, veh with mifepristone vs high dose CORT with mifepristone *p* < 0.001, post hoc Tukey test) (Fig. 6 H). The overall effect of mifepristone was not significant (*F*(1,47) = 1.88, *p* = 0.178), and the interaction of mifepristone and CORT treatment was not detected with 24 h exposure (*F*(2,47) = 0.88, *p* = 0.423), suggesting that the clear suppressive effect of CORT on *Pvalb* expression was not mediated by GRs.

**Fig. 7:**
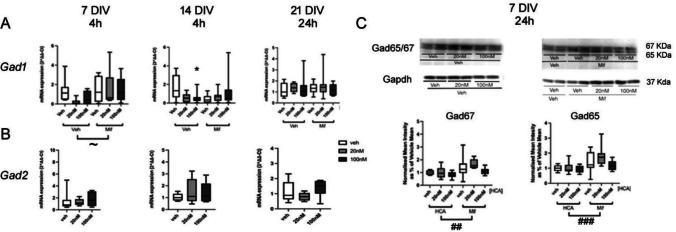
**A**, **B**: mRNA expression of *Gad1* (A) and *Gad2* (B) after 4 h (left and centre graphs) and 24 h (right graph) exposure to low dose (20 nM) or high dose (100 nM) CORT at 7, 14 and 21 DIV. *n* = 7–8/group (*Gad1*), 6–8/group (*Gad2* 7 DIV), 9–11/group (*Gad2* 14 DIV) or 12–14/group (Gad2 21 DIV). **C**: Immunoblotting with anti-Gad65/67 and anti-Gapdh antisera at 7 DIV (24 h) after CORT and mifepristone (20 nM) treatment (*n* = 96 in total: veh: veh = 16, low dose CORT = 16, high dose CORT = 16; Mifepristone: veh = 16, low dose CORT = 16, high dose CORT = 16). *p < 0.05 vs corresponding vehicle group (post hoc Fisher’s test); ^~^*p* = 0.053, ^##^*p* < 0.01, ^###^*p* < 0.001, overall effect of mifepristone vs corresponding vehicle groups, ANOVA. Boxes show median and interquartile range, with whiskers from minimum to maximum

*GAD1* expression is also robustly found to be decreased in PFC in schizophrenia [15, 16]. When tested either in the absence or presence of mifepristone at 7 DIV, the expression of *Gad1* was not significantly changed after 4 h CORT exposure (*F*(2,40) = 0.93, *p* = 0.406) (Fig. 7A). Equally, no changes were observed after 24 h exposure (Supplementary figure S7). However, the overall effect of mifepristone after 4 h exposure approached significance (*F*(1,40) = 4.01, *p* = 0.053), suggesting a possible suppression of *Gad1* expression by GCs in the culture medium that is relieved in the presence of mifepristone. There was no significant interaction between CORT and mifepristone exposure (*F*(1,40) = 4.01, *p* = 0.237). At 14 DIV, after 4 h exposure, no significant overall change in *Gad1* mRNA levels was found for either CORT (*F*(2,47) = 0.22, *p* = 0.803) or mifepristone (*F*(1,47) = 0.19, *p* = 0.661) (Fig. 7A). However, there was a significant interaction of CORT and mifepristone (*F*(2,47) = 0.823, *p* = 0.038), with the reduction of mRNA levels of *Gad1* after high dose (100nM) CORT exposure relative to vehicle in the absence of mifepristone (*p* = 0.045, Fisher post hoc test) (Fig. 7 A). At 21 DIV, with 24 h exposure, no effect of either CORT (*F*(2,46) = 0.71, *p* = 0.496) or mifepristone (*F*(1,46) = 0.21, *p* = 0.647) was observed, with no significant interaction between the two treatments (*F*(2,46) = 0.23, *p* = 0.798) (Fig. 7A).

In contrast to the effects of CORT on *Gad1* expression, no changes were detected in *Gad2* (GAD65) mRNA expression following CORT exposure at 7, 14 or 21 DIV at 4 or 24 h (*F*(2,23) = 0.38, *p* = 0.687 (4 h); *F*(2,23) = 0.33, *p* = 0.723 (4 h); *F*(2,20) = 0.73, *p* = 0.495 (24 h) respectively) (Fig. 7B, Supplementary figure S7).

Protein levels were also measured based on the mRNA alterations, at 7 and 14 DIV after 24 h exposure. At 7 DIV, there were no significant changes in Gad67/65 with 24 h CORT exposure, although there was a slight trend towards decreased expression (Gad67: 24 h: *F*(2,47) = 3.15, *p* = 0.053; Gad65: 24 h: *F*(2,47) = 2.61, *p* = 0.085) (Fig. 7 C). However there was a significant overall effect of mifepristone on Gad65/67 protein levels (Gad67: 24 h: *F*(1,47) = 12.72, *p* = 0.001; Gad65: 24 h: *F*(1,47) = 13.68, *p* = 0.001), with increased expression at 24 h. There were no significant interactions of CORT and mifepristone detected in Gad67/65 (Gad67: *F*(2,47) = 0.72, *p* = 0.493; Gad65: 24 h: *F*(2,47) = 2.61, *p* = 0.085) (Fig. 7 C). At 14 DIV, neither CORT nor mifepristone had any significant effect on *Gad1*/Gad67 or *Gad2*/Gad65 protein levels after 24 h exposure (Supplementary Figure S8). These results suggested that CORT had little overt effect on *Gad1*/Gad67 protein levels; however at 7 DIV, but not later in development, mifepristone exposure revealed a basal suppression of both Gad67 and Gad65 protein, and *Gad1* mRNA, by basal levels of GCs in the culture medium.

### Effect of Collagen 3 (GPR56/97 Agonist) and Aldosterone (MR Agonist)

The previous results showed that glucocorticoids could regulate the expression of PNN components, and the changes of *Has3*,* Gad1*, and *Pvalb* at 7 DIV could be reversed by the selective GR antagonist, mifepristone. However, the expression of other PNN components which were significantly regulated by glucocorticoids was not reversed by mifepristone. In this case, the effect might be activated via other GC targets rather than via GRs. Therefore, GPR56/GPR97 (ADGRG1/ADGRG3) were considered as a potential alternative pathway for the GC effect, as GCs are agonists at these receptors (GPR97/ADGRG3) [31], collagen 3, an agonist of GPR97 and GPR56 [34–36, 44] (considering the similarity between GPR97 and GPR56 at the binding site, they are likely to share the same ligands) [45] was tested on PNN component mRNAs which were affected by GCs but were not sensitive to mifepristone reversal (7 DIV: *Ncan*, *TnR*, 14 DIV: *Bcan*, *Vcan*, *Hapln4*, *Gad1*).

**Fig. 8:**
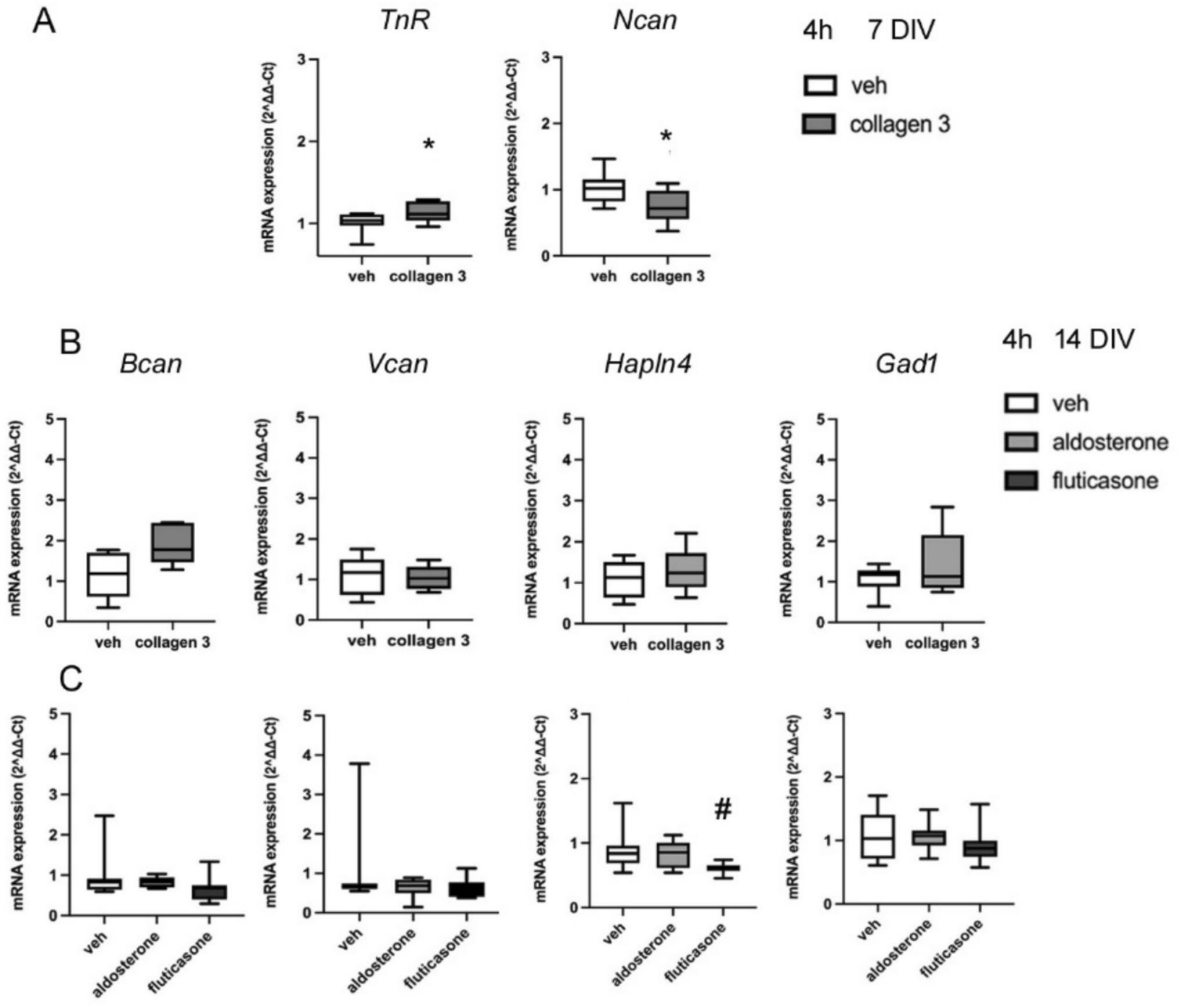
**A** mRNA expression of *TnR* and *Ncan* at 7 DIV after 4 h collagen 3 (75 nM) treatment (*n* = 23 in total, veh = 12, collagen 3 treated samples = 11). **B** mRNA expression of *Bcan*, *Vcan*, *Hapln4* and *Gad1* at 14 DIV after 4 h collagen 3 (75 nM) treatment. C. mRNA expression of *Bcan*, *Vcan*, *Hapln4* and *Gad1* at 14 DIV after 4 h treatment with aldosterone (100 nM) or fluticasone (50 nM) (*n* = 22 in total, Veh = 6, aldosterone treated samples = 8, fluticasone treated samples = 8). *p < 0.05 vs vehicle group, ANOVA; ^#^*p* = 0.024 vs vehicle group, Mann Whitney U test. Boxes show median and interquartile range, with whiskers from minimum to maximum

The mRNA expression of *TnR* was significantly upregulated by collagen 3 treatment for 4 h at 7 DIV (*p* = 0.020, *F*(1,23) = 6.33) (Fig. 8 A). *Ncan* mRNA expression was downregulated by collagen 3 treatment for 4 h (*p* = 0.006, *F*(1,23) = 9.29) (Fig. 8 A) at 7 DIV. The results indicated that collagen 3 could increase *TnR* mRNA expression and suppress *Ncan* mRNA expression rapidly. The former effect is opposite to the effect of GCs, but the effect on *Ncan* expression is similar, raising the possibility that both collagen 3 and GCs could be acting via the same (non-genomic) GPCR-mediated mechanism in this case.

In addition, the mRNA levels of *Bcan* (*p* = 0.071, *F*(1,10) = 4.20), *Vcan* (*p* = 0.985, *F*(1,10) = 0.00), *Hapln4* (*p* = 0.528, *F*(1,10) = 0.43) and *Gad1* (*p* = 0.434, *F*(1,10) = 0.67) were not affected by collagen 3 at 14 DIV, suggesting that the mRNA levels of *Bcan*, *Vcan*,* Hapln4* and *Gad1* were not affected through an action of glucocorticoids binding to GPR56/97 (Fig. 8 B).

In order to explore the mechanisms involved in these non-GR actions further, we compared the effects of the selective MR agonist aldosterone with the selective (no MR actions) GR agonist fluticasone. The mRNA expression of *Bcan* (*p* = 0.938; *F*(2,22) = 1.77), *Vcan* (*p* = 0.440; *F*(2,22) = 1.02), *Hapln4* (*p* = 0.887; *F*(2,22) = 3.30) and *Gad1* (*p* = 0.0.981; *F*(2,22) = 0.68) remained unchanged after exposure to aldosterone at 14 DIV for 4 h (Fig. 8 C). In terms of the effect of fluticasone, there was a decreasing tendency of *Hapln4* mRNA expression (*p* = 0.060; *F*(2,22) = 1.77; Veh vs fluticasone, *p* = 0.024, Mann Whitney U test) (Fig. 8 C), suggesting an inhibitory effect of fluticasone mediated by GRs. However, there was no significant change for *Bcan* (*p* = 0.201; *F*(2,22) = 1.77), *Vcan* (*p* = 0.460; *F*(2,22) = 1.02) or *Gad1* (*p* = 0.636; *F*(2,22) = 0.68) expression (Fig. 8 C). These results indicated that MRs were almost certainly not the mediators of the effects of CORT on the expression of these genes. Further, the clear lack of effect of fluticasone in the cases of *Vcan* and *Gad1* provided additional evidence that GRs are also not involved in these effects.

### No Evidence for GR-Mediated mRNA Decay Involvement

The GRs in the cytoplasm reportedly can bind directly to RNA, and GCs can activate the RNA-bound GR, leading to mRNA degradation, defined as the GC-mediated mRNA decay (GMD) pathway [46]. However, no alteration in the rate of *Bcan* or *Hapln4* mRNA degradation was detected after exposure to 20 nM CORT (Supplementary figure S9).

### Glucocorticoids Suppress Proteasome Activity

The effects on protein levels detected were sometimes consistent with the mRNA changes (e.g. *Has3*, *Gad1*) and sometimes unrelated to them (e.g. elevated TnR protein levels at 7 DIV after GR antagonism). We explored the hypothesis that GCs might exert rapid non-genomic post-transcriptional effects on protein expression via modulating proteasome activity. To test whether the glucocorticoids affect proteasome activities, neuronal cultures at 14 DIV, treated with hydrocortisone for 4 h, were measured with a fluorogenic proteasome assay with 3 different proteasome substrates related to the 3 proteasome activities, including chymotrypsin-like activity, caspase-like activity, and trypsin-like activity. All activities were completely inhibited by 10 mM MG132 (data not shown).

**Fig. 9:**
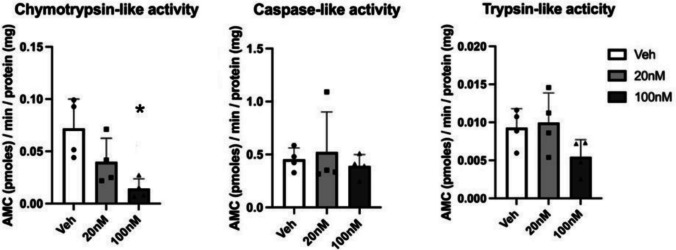
20S proteasome activities (chymotrypsin-like, caspase-like and trypsin-like activity) from cortical cultures at 14 DIV treated with veh (distilled water), 20 nM or 100 nM CORT. *n* = 4/group. *p < 0.05 vs vehicle group (post hoc Tukey test). Results are shown as mean ± s.e.m. with individual data points

The results of the proteasome assays showed that low and high doses of CORT tended to inhibit the proteasome activities. The chymotrypsin-like activity could be inhibited by both low dose and high dose CORT compared to the vehicle group (Fig. 9); however, only the inhibition by high dose CORT was statistically significant (*F*(2,11) = 7.18, *p* = 0.011 veh vs high dose CORT; *p* = 0.144 veh vs low dose CORT, post hoc Tukey test). However, no apparent effect of low or high dose CORT on caspase-like activity was observed (*F*(2,11) = 0.32, *p* = 0.910 veh vs low dose CORT; *p* = 0.923 veh vs high dose CORT, post hoc Tukey test) (Fig. 9). Moreover, only very low activity was observed using the substrate which detected the trypsin-like activity (data not shown). Although the inhibition tendency was shown with high dose CORT exposure, the inhibition was not significant (*F*(2,11) = 2.65, *p* = 0.947 veh vs low dose CORT; *p* = 0.219 veh vs high dose CORT, post hoc Tukey test).

A summary of the effects of CORT on PNN gene expression is provided in Table 1.

### CORT Effects on PNN Structure

While PNNs are not detectable at 7 DIV, the widespread and diverse suppressive effects of GCs on the genes encoding components of PNNs that we have observed at 14 DIV strongly suggest that GC exposure is likely to have detrimental effects on PNN formation at this stage, but potentially not at 21 DIV. To clarify the impact of glucocorticoids on dendritic and structural formation of PNNs, WFA-labelling was monitored in cultured cells exposed to the same treatments.

**Fig. 10:**
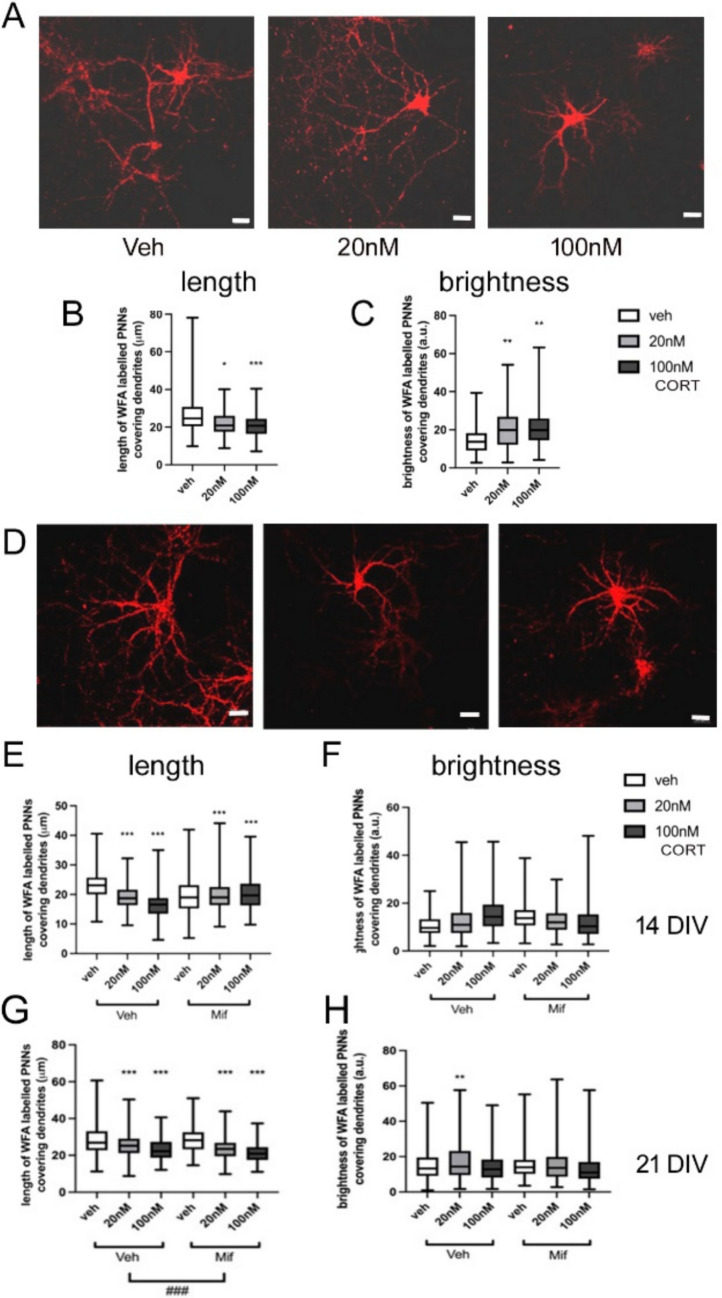
Effect of 24 h glucocorticoid exposure on WFA-labelled PNNs in cultured neurons at 14 or 21 DIV. **A** Representative images of WFA-labelled PNNs around cultured neurons at 14 DIV after 24 h exposure to vehicle, 20 nM or 100 nM CORT. **B** Length of dendrite covered by PNN, and **C** brightness intensity of WFA staining (Veh: 270 dendrites in 20 cells nested in 3 different slides with 2 cultures, 20 nM: 166 dendrites in 20 cells nested in 3 different slides with 2 cultures, 100 nM: 199 dendrites in 16 cells nested in 3 different slides with 2 cultures). **D** Representative images of WFA-labelled PNNs around cultured neurons at 14 DIV after 24 h exposure to vehicle, 20 nM or 100 nM CORT in the presence of mifepristone (20 nM). **E** Length of dendrite covered by PNN, and **F** brightness intensity of WFA staining (Veh: veh: 139 dendrites in 13 cells nested in 3 different slides with 2 cultures, 20 nM: 275 dendrites in 17 cells nested in 3 different slides with 2 cultures, 100 nM: 164 dendrites in 15 cells nested in 3 different slides with 2 cultures; Mif: veh: 290 dendrites in 21 cells nested in 3 different slides with 2 cultures, 20 nM: 182 dendrites in 16 cells nested in 3 different slides with 2 cultures, 100 nM: 177 dendrites in 14 cells nested in 3 different slides with 2 cultures). **G**,** H** Effects on PNNs at 21 DIV after 24 h treatment**—G** length of dendrite covered by PNN, and **H** brightness intensity of WFA staining (Veh: veh: 360 dendrites in 32 cells nested in 3 different slides with 2 cultures, 20 nM: 263 dendrites in 20 cells nested in 3 different slides with 2 cultures, 100 nM: 265 dendrites in 20 cells nested in 3 different slides with 2 cultures; Mif: veh: 165 dendrites in 20 cells nested in 3 different slides with 2 cultures, 20 nM: 297 dendrites in 18 cells nested in 3 different slides with 2 cultures, 100 nM: 291 dendrites in 19 cells nested in 3 different slides with 2 cultures). ^###^*p* < 0.001 ANOVA main effect mifepristone vs vehicle group **p < 0.01, ***p < 0.001 post hoc Tukey test vs corresponding vehicle control group. Scale bars represent 20 mm. Boxes show median and interquartile range, with whiskers from minimum to maximum

At 14 DIV, exposure to 20 nM or 100 nM CORT resulted in decreased length of PNNs covering dendrites after 4 h (*F*(2,946) = 56.11, *p* < 0.001) (data not shown), with reduction of mean length from 29.3 µm to 23.4 µm with 20 nM CORT (Tukey post hoc tests, veh vs 20 nM CORT, *p* < 0.001) and to 21.7 µm with 100 nM CORT exposure (Tukey post hoc tests, veh vs 100 nM CORT, *p* < 0.001). Length of dendrites covered by PNNs also decreased after 24 h (*F*(2,673) = 14.89, *p* < 0.001) CORT exposure (Fig. 10 A, B), with reduction of mean length from 25.3 µm to 21.0 µm with 20 nM CORT (Tukey post hoc tests, *p* < 0.001) and to 23.4 µm with 100 nM CORT exposure (Tukey post hoc test, *p* = 0.044). Similarly, the brightness intensity of WFA-labelled PNNs covering dendrites also decreased after 24 h (*F*(2,673) = 14.89, *p* < 0.001) with both 20 nM and 100 nM CORT treatment, from 23.09 a.u. to 20.13 a.u. and 16.9 a.u., respectively (*p* = 0.024 Veh vs 20 nM Tukey post hoc test; *p* < 0.001, Veh vs high dose, Tukey post hoc test) (Fig. 10 A, C); however, no significant changes in brightness of staining were found after 4 h (*F*(2,946) = 1.12, *p* = 0.327) treatment (data not shown). The changes observed were not reversible with mifepristone (Fig. 10 D-F).

Moreover, at 21 DIV, there was still an overall effect of CORT and of mifepristone on the length of PNN-covered dendrites after 24 h treatment (*F*(1,1580) = 47.13, *p* < 0.001). The interaction of CORT treatment and mifepristone was not quite significant (*F*(2,1580) = 2.81, *p* = 0.060), but with a decreasing tendency in mean length of PNNs covering dendrites from 22.5 µm to 19.0 µm after 24 h 100 nM CORT with co-treated mifepristone (Tukey post hoc tests, *p* < 0.001). However, no overall effect of mifepristone on the brightness intensity of WFA-labelled PNNs covered dendrites was found after 24 h CORT and mifepristone treatment (*F*(1,1580) = 47.13, *p* = 0.576) (Fig. 4 G, H); however, there was a marginally significant interaction of CORT and mifepristone treatment (*F*(2,1580) = 3.06, *p* = 0.047) with slightly increased brightness intensity of WFA-labelled PNNs after 100 nM CORT and mifepristone treatment (Tukey post hoc test, *p* = 0.046).

Hence we observe robust decreases in the length of proximal dendrite covered by PNN after CORT exposure, at both 14 and 21 DIV. These effects on PNN structure do not appear to be mediated via GRs.

Since loss of TnR reportedly reduces the number of dendrites that are covered proximally by PNNs [47], we also checked this parameter. However, no change in the number of WFA-labelled dendrites/cell was observed after CORT or mifepristone exposure (Supplementary figure S10).

## Discussion

A strength of this study is that the expression of all the genes encoding core PNN components has been monitored in parallel. Hence the relative effects on the different genes can be interpreted with confidence. Our initial expectation was that we might identify one or two components of the PNN where gene expression is modulated by CORT levels. Instead, we found a widespread and complex regulation of multiple PNN component genes, via diverse mechanisms, and suggesting a prominent role for GCs in modulating PNN gene expression, but potentially limited to early in development. The diminishing evidence for GC regulation of these genes from 7 to 21 DIV suggests that these gene expression mechanisms are very important while PNNs are forming and stabilising, but are much less important once they have attained a mature configuration.

Only for the phosphacan/*Ptprz1* gene was there a complete lack of evidence for regulation by GCs. Even in this case, GC modulation may be just hidden rather than absent. The exons encoding the phosphacan protein are also present in longer mRNAs encoding the full length Ptprz1 protein, and so any regulation specific for the phosphacan isoform might not be evident among the total transcripts, although a genomic action should still be evident, as, occurring prior to mRNA splicing, it should affect all protein isoforms.

### Mechanisms of GC Regulation of PNN Gene Expression

Basal human serum cortisol concentrations are in the range of 150 to 350 nM, although around 80% is not free in solution, but is bound to cortisol-binding globulin [48–51], leaving free concentrations of around 30–70 nM, although there is also marked circadian oscillation. Under conditions of high psychological stress, levels can rise to over 1000 nM [52–54]. Similarly, in mice, resting total corticosterone levels are around 100–350 nM (free concentrations ~ 20–70 nM), rising to 1000–3000 nM (free concentrations ~ 200–600 nM) during stress [55]. During pregnancy, where mothers are not stressed, foetal cortisol levels have been estimated to be around 55nM [23]. Neuronal survival in culture relies on the presence of basal levels of GCs. Neurobasal medium with B27 supplement (which does not contain cortisol- binding globulin) contains CORT concentrations of around 50–70 nM [56–60], in fact very similar to basal free GC concentrations in a non-stressed condition. Accordingly, we selected 2 concentrations of CORT to be added as experimental manipulation in this study, to represent both physiological (20 nM) and pathological (100 nM) levels of stress response. Resting GC concentrations will be fully activating MRs, whereas increasing concentrations under stress will additionally stimulate increasing proportions of GRs (and both the 20 nM and 100 nM concentrations will also activate GPR56/97) [31, 61–64].

In fact, where we observed effects of 100 nM CORT, we generally also observed the effect with the lower concentration. At 7 DIV, these effects were mostly mediated by GRs, as indicated by sensitivity to mifepristone. Where a significant effect was detected with the higher but not the lower concentration (as in suppression of *Gad1* and *Has3* expression), the same trend was observed with the lower concentration, consistent with dose dependency at a single receptor. These effects at 7 DIV, affecting many PNN component genes, reveal a widespread sensitivity of PNN component gene expression to GC levels at this developmental time, mediated by GRs.

At 7 DIV, CORT suppressed *Ncan* and *TnR* expression, and mifepristone enhanced *Acan* expression and further suppressed *Ncan* and *TnR* expression. Thus basal GC levels are tending to promote *Ncan* and *TnR* expression relative to *Acan* expression, and there are additional non-GR-mediated GC actions to dampen down *Ncan* and *TnR* expression. Modulation of *Has* gene expression is prominent at this developmental stage—CORT suppressed *Has* 1, 2 and 3 expression via GRs, whereas at 14 DIV, this effect was not evident, but mifepristone elevated *Has* 1 and 2 expression. This implies that GCs are still acting to reduce *Has* 1 and 2 expression through GR activation at 14 DIV, but that the effect has become more sensitive, so that the low GC concentrations in the culture medium are now effective. A powerful GC/GR-mediated suppression of *Has* 1, 2 and 3 mRNA has been noted in peripheral cells [65–67].

Effects of mifepristone alone suggest alleviation of actions of basal levels of glucocorticoids in the culture medium, which as noted above, are sufficient to produce some activation of GRs. However, the culture medium also contains progesterone, and mifepristone is a high-affinity antagonist at progesterone receptors as well as GRs. Hence the possibility of mifepristone acting by antagonising the effects of ambient progesterone cannot be completely excluded.

Apart from the elevated levels of *Has1 *and *Has2* mRNAs induced by mifepristone at 14 DIV, decreased *Bcan* expression was also observed, despite the lack of GC modulation at 7 DIV, and suggesting heightened mRNA levels at 14 DIV due to basal GC levels. The other effect detected at 14 DIV was a pronounced suppression of *Vcan* and *Hapln4* expression by CORT, where there also appeared to be increasing sensitivity to GCs, as no significant suppression was detected at 7 DIV; although, for the effect on *Vcan* mRNA levels at 14 DIV, GRs appeared not to be involved. Conversely, mRNA levels for *Ncan*, *TnR* and *Gad1* had now become insensitive to GCs.

While reduced expression of *Bcan*, *Ncan* and *Vcan* mRNA expression following GC exposure appears not to have been previously reported in the CNS, both the *Acan* and *Ncan* gene promoters contain GR-response elements [68, 69], and GC suppression of *Vcan* and *Acan* expression has been noted in peripheral cells [65, 70, 71]. Interestingly, prenatal exposure to GCs downregulates peripheral tissue *Acan* expression [72], suggesting that early developmental exposure can have lasting effects in offspring.

There is some existing evidence suggesting suppression of *Ncan* expression by stress or GC exposure. Liu et al. [73] reported GC-induced downregulation of expression *of Ncan* in astrocyte cultures (1 DIV) over 48 h, partially mifepristone-sensitive, and intracerebral dexamethasone also reportedly decreased *Ncan* immunoreactivity [74]. Chronic stress in adolescence or in adulthood in rodents also seems to suppress cortical or hippocampal *Ncan* expression [13, 75]. Adult rodents exposed to stress show downregulation at the protein level of hippocampal Bcan, Ncan, Ptprz1, TnR and Hapln1 but not Acan [13] and PFC Acan but not Bcan [14].

The suppression of *Ncan* expression by GCs at 7 DIV was not attenuated by mifepristone, but a similar suppression was detected following exposure to collagen 3, an agonist at Gpr56 and Gpr97. It is interesting to note that *Ncan* appears to be synthesised primarily by astrocytes and glutamatergic projection neurons [8, 76, 77] (there will be a small proportion of astrocytes, 3–5%, present in our neuronal-enriched cultures), and Gpr56 is expressed predominantly in astrocytes (but not in glutamatergic projection neurons [76–78]). Hence it is possible that GCs can act via GRs to produce a generalised enhancement of Ncan expression, whereas GC activation of Gpr56 can reduce *Ncan* expression specifically in astrocytes.

GCs are reported to exert a post-transcriptional regulation of gene expression by GC/GR complexes affecting mRNA stability [46]. However, we did not obtain any evidence to support this mechanism of action for the regulation of PNN component gene expression. Indeed, for many GC-induced changes in PNN component (suppression of *Bcan*,* Vcan* and *TnR* mRNA levels), we were unable to pinpoint the mechanisms of GC action. GRs appeared not to be involved (since mifepristone had little ability to attenuate the effects), and neither did Gpr56/97 (as reflected in the inability of collagen 3 to replicate the effect), but the effects seemed rapid, in that they were detectable within 4 h, and so are likely to be non-genomic in nature.

### Effect on PNN Structure

Compromised PNN formation (brightness of WFA staining) in the cortex and hippocampus after exposure to prenatal or neonatal stress is well documented in rats and mice [10–12, 79, 80]. Most studies using mouse hippocampal or cortical cultures concur that at 14 DIV, PNNs are almost mature, with a net-like structure covering somata and proximal dendrites, and from then on the morphology changes little [81–83]. Our observations were similar. This report may be the first demonstration that GCs modify PNN structure directly, and hence may be the mediators of the effects of stress on PNNs, although, while establishing a potential mechanism, confirmation that this action can occur in vivo is still needed.

GC exposure caused a small but robust reduction in the length of PNNs ensheathing the proximal dendrites, not involving GRs, and a more slowly developing increase in the intensity of WFA staining, likely to be mediated by GRs. The different mechanisms involved suggest that the increased brightness of WFA staining is not simply a result of compaction of a constant amount of PNN into a smaller volume. At 14 DIV, the magnitude of the changes in PNN structure might be considered smaller than expected, considering the quite profound changes in gene expression observed with GC exposure. However, PNNs seem to be quite robust to altered expression of component genes. While loss of Acan, phosphacan, TnR or Hapln4 compromises PNN structure [47, 84–92], PNNs appear unaffected even in the complete absence of Bcan, Ncan or Has3 [93–95].

The suppression of *Bcan*, *Vcan* and *Hapln4* gene expression by GCs at 14 DIV was not mifepristone-sensitive, and equally the decrement in PNN structure was also not mifepristone-sensitive. Hence the altered WFA staining could be due to alterations in PNN component gene expression. Equally, no changes in PNN component gene expression were detected from GC exposure at 21 DIV, yet altered WFA staining characteristics are still observed. We have not systematically profiled PNN component protein expression at 21 DIV, so post-transcriptional actions of GCs may be responsible. For example, it has been suggested that Acan levels are partly controlled by post-translational modifications [35, 36], so if these processes were modulated by GCs, a subtle but rapid effect on PNN structure might be evident.

### Modulation of GABAergic Interneuron Genes

Expression of both *Has3* and *Hapln4* (suppressed by GCs at 7 and 14 DIV) appears to be markedly enriched in Pvalb+ve cells as compared to other cortical neurons [76]. The regulation of *Has3* and *Hapln4* mRNA levels (and protein levels for Has3) by GCs might suggest a particular action of GCs on Pvalb+ve cells.

Elevated GC levels suppressed *Gad1* expression (7 and 14 DIV) and *Pvalb* expression (21 DIV), and at 7 DIV, blockade of GRs with mifepristone elevated expression of both *Gad1* and *Pvalb*. These 2 GABAergic interneuron genes, both of which show reduced expression levels in schizophrenia, are clearly sensitive to elevated GC levels. The action on *Gad1* expression at 7 DIV and 14 DIV appeared to involve GRs, whereas the action on *Pvalb* expression at 21 DIV, when PNN component genes were apparently resistant to GC actions, involved another mechanism.

These results are consistent with some previous observations. There are reports that chronic stress prenatally [10, 96–98], neonatally [99] and in adult rodents [100, 101] decrease cortical *Pvalb* expression, although increased expression after adult stress has also been reported [102]. High GC concentrations for 72 h also reportedly suppress *Gad1* (but not *Gad2*) expression in 10 DIV cultured cortical neurons [100], and prenatal GC exposure in vivo reduces offspring *Pvalb* expression in the hippocampus [103]. Hence our data, along with previous reports, seem clear in identifying Pvalb+ve cells potentially as direct targets of GCs, and hence illustrate a possible mechanism for inducing gene expression changes relevant to schizophrenia.

### Proteasome

Proteasome inhibition leads to elevated cellular levels of proteins which are proteasome substrates. The modulation of neuronal proteasome activity by GCs has not previously been reported. There are some hints of proteasome modulation by GCs from peripheral cells, although for stimulation rather than inhibition. In hepatocytes, dexamethasone stimulates protein degradation within 4 h [Hopgood et al., 1980], and similarly in muscle cells GCs accelerate protein degradation via ubiquitin pathways [104]. There is a single report, in thymocytes, of physiologically relevant concentrations of dexamethasone suppressing chymotrypsin-like and caspase-like, but not trypsin-like, proteasome activity within 3 h [105].

Here we report an inhibition of neuronal proteasome activity that is specific for chymotrypsin-like activity as compared to caspase-like (post-glutamate peptide hydrolase-like) activity (trypsin-like activity was very low, and it might be more difficult to observe suppression of activity). It should be noted that this suppression of chymotrypsin-like activity by CORT was demonstrated at 14 DIV. It seems likely that a similar action of glucocorticoids on the proteasome occurs at other developmental stages, but additional experiments would be needed to formally demonstrate this. Additionally, the effects of mifepristone at 7 DIV on TnR protein levels that were independent of mRNA changes were elevating rather than suppressing, which would rather imply that GCs could be enhancing TnR degradation. Hence effects on proteasome activity are unlikely to explain these observations.

## Conclusions

Perinatal trauma, which increases offspring schizophrenia risk [106, 107], elevates neonatal cortisol levels [108–111]. At this period, corresponding to our 7–14 DIV cultured neurons in mice, as PNNs form and stabilise, cortical *Ncan* and *Vcan* expression is declining, while *TnR* and *Pvalb* expression is increasing, in mice [85, 112–116], and in humans [117–120]. Our data suggest that inappropriately elevated cortisol levels at this time will be sufficient to disrupt the expression of PNN component genes at a critical time for PNN formation. We propose this mechanism as potentially contributing to the abnormal properties of Pvalb+ve interneurons in schizophrenia, although, as our data derive entirely from an in vitro culture system, in vivo confirmation will be required.

## Supplementary Information

Below is the link to the electronic supplementary material.

ESM 1 (DOCX 6.94 MB)

## Data Availability

All data are available from the corrresponding author upon reasonable request.
